# A novel nonsense mutation in exon 9 in the extracellular matrix protein 1 gene associated with lipoid proteinosis: A case report

**DOI:** 10.1177/2050313X19850359

**Published:** 2019-05-19

**Authors:** Feras M Ghazawi, Etienne Saint-Cyr Proulx, Fatemeh Jafarian

**Affiliations:** 1 Division of Dermatology, University of Ottawa, Ottawa, ON, Canada; 2 Hôpital de la Cité-de-la-Santé, Laval, QC, Canada; 3 Innovaderm Research Inc., Montreal, QC, Canada; 4 Division of Pediatric Dermatology, McGill University Health Center, Montreal Children’s Hospital, Montreal, QC, Canada

**Keywords:** Lipoid proteinosis, extracellular matrix protein 1, ECM1, exon 9, nonsense mutation

## Abstract

Lipoid proteinosis is a rare autosomal recessive genodermatosis that is caused by loss-of-function mutations in the extracellular matrix protein 1 gene. This study identifies a novel nonsense mutation in exon 9 of the extracellular matrix protein 1 gene associated with lipoid proteinosis, contributing to recent advances in our understanding of the molecular genetics underlying this disease. It is important to identify the mutations in the extracellular matrix protein 1 gene that are associated with lipoid proteinosis and how these affect protein function. Understanding the molecular basis for such genetic disorders may lead to novel therapeutic approaches for treating hereditary genodermatoses.

## Introduction

Lipoid proteinosis, also known as Urbach–Wiethe disease or hyalinosis cutis et mucosae, is a very rare autosomal recessive disorder with approximately 400 cases described. It is caused by a genetic defect, resulting in the deposition of amorphous hyaline material in the skin, mucosa, internal organs, and central nervous system.^1^ Clinically, lipoid proteinosis is typically characterized by hoarse voice due to mucosal thickening, laryngeal infiltration, and deposit accumulation in the vocal cords that collectively result in increased vocal fold mass and stiffness as well as irregularity and/or reduction in vocal fold mucosal wave.^2^ Lipoid proteinosis is also associated with skin thickening, fragility, poor wound healing capacity, development of hyper-, hypo-, or depigmented scarring and characteristic “moniliform blepharosis” beaded papules on eyelids and thickened mucosa of the tongue as well as neurological complications including seizures.

Lipoid proteinosis is caused by loss-of-function mutations in the extracellular matrix protein 1 gene (ECM1).^1^ The ECM1 gene is located on chromosome 1q21,^3^ next to the epidermal differentiation complex. It comprises 11 exons and encodes four splice variants.^4^ ECM1 gene products have been reported to be involved in extracellular matrix formation, cell adhesion, cell signaling, regulation of tissue differentiation and maturation, angiogenesis, and bone formation.^5–7^ However, a clear understanding of their functions remains to be established. More than 50 ECM1 mutations have been reported in the literature, with the majority of mutations (~50%) reported in exons 6 and 7.^8^ No effective therapy is available for this condition yet.

## Case report

Here we present two brothers aged 16 and 5 years at the time of presentation, who were referred to our clinic for diffuse cutaneous scarring. They presented with a long-standing history of blistering on the face, arms, trunk, and lower limbs during early childhood that had resolved with scarring. Both boys had developed profound hoarse voices within their first few months of life. Obstetrical and developmental histories were unremarkable. There was no history of loss of consciousness, headache, or epilepsy. The elder brother was attending high school in good standing. Our patients were the only affected individuals in a sibship of six. The parents were first cousins, and both originated from the Hazara region of Pakistan. Two paternal uncles and a paternal aunt reportedly shared similar cutaneous and vocal symptoms. No neuropsychiatric abnormalities were reported in the patients or their family.

**Figure 1.**
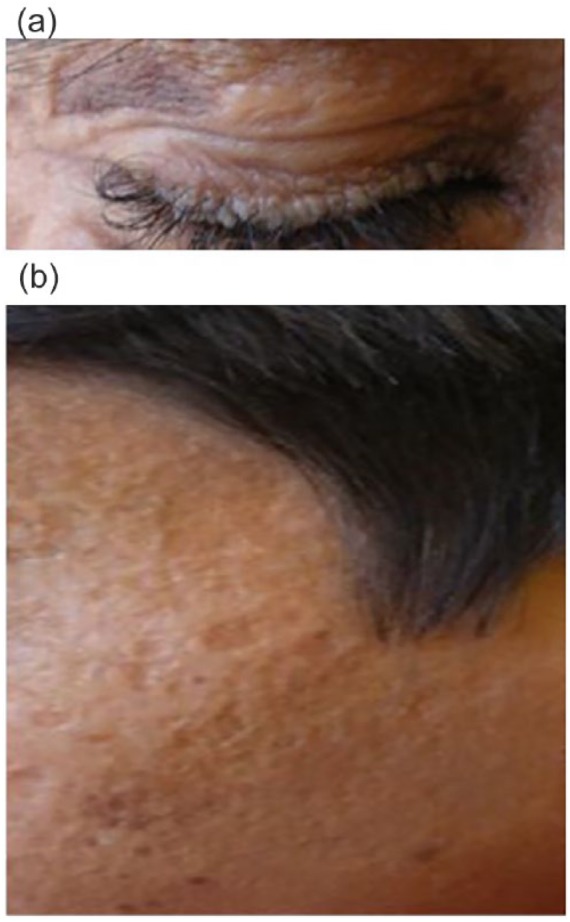
Clinical phenotype in the children with lipoid proteinosis, confirmed by genetics testing to have a nonsense mutation in exon 9 of the ECM1 gene, demonstrating the following clinical manifestations: (a) row of beaded papules along the eyelid margins, resembling a string of pearls; this is termed moniliform blepharosis; (b) pox-like atrophic scarring on the face on a waxy, thickened, yellowish appearance of skin.

Physical examination of both siblings was notable for confluent pock-like scars on the face, trunk, upper extremities, and small beaded papules lining the eyelids (Figure 1). The patients’ voices were remarkably hoarse. Examination of the oral mucosae revealed normal appearing teeth and a short tongue with infiltrated frenulum in the younger brother. Diffuse waxy skin thickening and verrucous papules were noted over the extensor surfaces of the upper limbs of the elder brother. Neurological exams were within normal limits.

Investigations including complete blood count, serum biochemistry, electroencephalography, and laryngoscopic examination were all within normal limits. Ophthalmologic evaluation revealed mild myopia in the younger brother. No intracranial anomalies were detected on magnetic resonance imaging and computed tomography scans of the brain. Skin biopsies were performed and revealed Periodic acid–Schiff positive diastase-resistant hyaline deposition in the basement membrane, surrounding blood vessels, adnexal epithelia, and the dermo-epidermal junction. Electron microscopy showed thickened vessel walls with embedded pericyte extension within concentric arrays of basement membrane-like material and irregular concentric reduplication at the epidermal basement membrane.

After receiving informed consent, genetic analysis was performed on saliva specimens obtained from both patients and revealed a homozygous G>T transversion (c.1387G>T) predicted to result in a premature stop codon (p.Glu463X) in exon 9 of the ECM1 gene. Our patients were started on acitretin at 0.5 mg/kg/day. The treatment was well tolerated but no objective or subjective improvement of the skin lesions or hoarseness was noted after 6 months and the treatment was discontinued.

## Discussion

Erich Urbach, an Austrian dermatologist, and Camillo Wiethe, an Austrian otorhinolaryngologist, first described “lipoidosis cutis et mucosae” in 1929.^9^ Nearly a century later, about 400 cases of lipoid proteinosis have been described in the literature. Epidemiologic data for this rare disease is understandably scarce. The Namaqualand region of South Africa is the area of the world that has the highest prevalence of lipoid proteinosis cases. Genetic studies performed on all the cases from South African yield the unique c.826C>T mutation, suggesting a founder effect in this population.^10,11^ Notably, at least 25 patients, including the one identified in the present report, have been described with ECM1 mutations in cases from the Hazara and Rawalpindi districts in Pakistan.^12–14^ Javeria and colleagues^12^ reported five separate cases from different families from the Hazara region, but with no genetic analysis. Furthermore, Nasir et al.^13^ reported 17 living members of a six-generation family from the adjacent Rawalpindi district in Pakistan, affected by lipoid proteinosis, and genetic testing revealed insertion in exon 8 of the ECM1 gene. In addition, two siblings from the Rawalpindi district were diagnosed with lipoid proteinosis with mutations in exon 6.^14^ Other patients from other regions from Pakistan were also reported.^15^ The elevated number of cases from this region with regard to its relatively limited population raises the question whether a founder effect might be present in this population.

It has been reported that the majority (~50%) of mutations reported in the ECM1 gene were on exons 6 and 7.^8^ Mutation in exon 6 was associated with severe phenotype while mutations in exon 7 were associated with a mild form.^8^ Only three mutations in the initial first two exons or in the first intron were described.^16^ The present study highlights a novel mutation in exon 9. To our knowledge, only one additional study described a loss-of-function mutation in exon 9 of the ECM1 gene in a patient with lipoid proteinosis.^17^

Many systemic therapies have been reported for lipoid proteinosis including chloroquine phosphate,^18^ corticosteroids,^19^ dimethyl sulfoxide,^20^ D-penicillamine,^21^ and etretinate.^22^ However, none of these treatments have shown clear and lasting benefits. More recently, acitretin has been suggested to improve skin and hoarseness.^23–25^

In summary, we report a novel nonsense mutation (c.1387G>T) in exon 9 of the ECM1 gene associated with lipoid proteinosis. Although previous studies have reported improvement of the skin lesions or hoarseness with acitretin, no improvement was observed in our case. As it is the case for all rare diseases, large randomized studies are not feasible and the search for an effective therapeutic option relies on trial and error. Emerging treatments including Er:YAG (erbium-doped yttrium aluminum garnet) laser to ablate disfiguring lesions,^26^ and a combination regimen of fractional carbon dioxide and non-ablative radio frequency were recently reported efficacious in the cosmetic treatment of the scars by this disease.^27^ Further research into the pathophysiology of lipoid proteinosis will hopefully provide further insight to guide future therapeutic trials.
